# Devitalized (Dead) Teeth*Extract from a paper published in the June, 1918, *Journal of N. D. A.*, with tables illustrating cases treated.

**Published:** 1918-07

**Authors:** Josef Novitzky


### DEVITALIZED (DEAD) TEETH *

BY JOSEF NOVITZKY, D.D.S.

At the present time dental students are particularly interested in methods of treating devitalized teeth. They are taught that devitalized teeth should be retained in the jaws to serve as organs of mastication. They are led to believe that the dentin of a devitalized tooth can be so sterilized as to prevent suppurative and putrefactive changes from taking place in the tooth and in the surrounding parts.

Hence dentists freely devitalize teeth, treat the devitalized teeth by various methods, and send the patients away assured that the devitalized teeth are practically as good as vital ones. There is no mention of the fact that devitalized teeth are apt to be harmful when retained in the jaws.

Physicians realize that many metastatic lesions are secondary to virulent pathogenic organisms in the jaws. To these organisms may often be traced asthma, septic bronchitis, pneumonia, skin eruptions, rheumatic conditions of joints, muscles, and nerves, heart and kidney lesions,

---

* Extract from a paper published in the June, 1918, *Journal of N. D. A.*, with tables illustrating cases treated.

chronic toxemias, bacteremias, and anemias. But dentists do not realize that virulent pathogenic organisms in the jaws are usually due to the retention of devitalized teeth. The conditions in the jaws can not be seen in the ordinary examinations of teeth; and the minds of dentists are on things other than the hidden pathogenic organisms of the jaws and the future health of the patients.

A typical case is the following: A woman had her breast removed seven years before. Two years after the removal there was recurrence of the growth. This was followed by operation. One year ago, an induration was noticed just above the clavicle on the affected side. There was also an indurated mass under the angle of the jaw on the operated side. This was thought to be malignant. The removal of the lower first and second molars with quite an extensive amount of carious process and also of septic bicuspids in the upper jaws, resulted in a complete disappearance of the indurations; resolution being encouraged with moist heat. The patient has also noticed that after the jaw surgery, the arm of the affected side no longer swelled and stiffened. This patient had almost decided to submit to a radical operation for a malignant growth.

The necessity of the prompt removal of dead teeth and necrosed bone from the jaws may be seen by the following case. Unobserved by the patient and by his physician, injury to the heart was undoubtedly being caused by conditions of the jaw. The cause of the injury was removed by operation; but the injury to the heart had been done. The removal of the cause was too late.

A patient, aged forty-seven years, sought my advice because of a toothache. The average case that presents is referred for aches and pains in remote parts that are thought to be due to dental origin. Roentgen ray examination indicated evidence of suppurative destruction in the apical region of the right upper first molar. Blood pressure examination showed a systolic pressure of 195 and a diastolic pressure of 140. Urinalysis showed casts and albumen. These findings were confirmed by the medical attendant.

The patient apparently was in the best of health and made no mention of illness or pain aside from the toothache. At operation on the upper molar the outer plate was dissected away from the buccal roots. This exposed a large cavity filled with offensive odorous granulations. A necrotic opening was found leading into the antrum. Granulations from the necrosed root protruded into the cavity. The unhealthy parts were thoroughly removed and the mouth wound sutured.

Bacterial growth from these granulations was reported by Dr. Knapp of St. Luke's Hospital, San Francisco, to be a pure strain of a short chain streptococcus of the viridans type.

Three weeks after operation the wound was nicely healed and the patient's weight had increased about seven pounds. Blood pressure examination showed a systolic pressure of 180 and a diastolic pressure of 125. Urinalysis was negative.

This marked improvement followed the jaw surgery, as no medication of any kind, outside of free drinking of mildly alkaline waters had been undertaken.

Some three months after the operation the patient was called upon to exert himself over a period of some days, during which he was on duty day and night with little sleep. While on duty he was taken ill with a severe pain in the heart and died a few hours later in spite of the best medical help.

Experience with similar cases has led to the conclusion that the injury to the heart had been caused by the condition of the jaw previous to the operation. Injury to parts of the body remote from the mouth is commonly due to the practice of devitalizing teeth, and treating and retaining dead teeth in the jaws.

Whether a medical examination of the body does or does not show deviations from the normal, injury of more or less degree undoubtedly takes place.

Devitalized teeth in which putrefactive changes have taken place should be removed from the jaws not by the ordinary "pulling" or extraction but by a surgical dissection.

Under local anesthesia the gums and the periosteum may be cut loose and stripped down along as much of the length of the jaw as is necessary to gain free space for operating. But generally it is sufficient to raise and pull back a triangular flap with the apex at the gingival margin of the affected tooth. Thus the outer plate of bone for the entire root length is exposed.

Then the outer plate of bone over the involved root is removed with the chisel so as to expose the cancellous bone with the tooth root in position. In many cases the root may be used as a guide in following up minute drainage ways into neighboring canals or cavities.

Now, since the region is blocked off by sterile sponges and hemorrhage is under control, specimens of granulation tissue and pus may be isolated.

If a cavity is found under the roots of the tooth, the tooth and the alveolar septum are removed with the chisel. The inner plate of the jaw is left intact except in a few cases in the upper jaw which involve the antrum. In such cases enough of the inner plate of the upper jaw is removed to allow the inner flap of mucous membrane and periosteum to be pulled over to meet the outer. Injury to the inner plate should be avoided whenever possible, because when both plates are removed, bone regeneration takes place more slowly in the inner plate than in the outer. Also some deformity may follow the removal of both plates.

Antrum incisions may be sutured immediately after the operation because drainage takes place through the natural ostium into the nose. Unless hemorrhage is excessive gauze packings are unnecessary. If the cavity must be irrigated, a cannula may be inserted through the flap from the mouth or through the thin plate of bone under the inferior turbinate. When antrum pathology is due to a dead tooth, the method of approach from the mouth allows direct vision and permits the opening to be extended with minimum shock to the patient. It retains the nasal wall intact and thus prevents the accumulation of mucous in the cavity through an unnatural opening from the nose.

The surgical dissection outlined above renders certain a clean removal of the diseased area, because it offers a clear view of the operative field. It results in little inconvenience and no damage to the patient. After the operation the alveolar process will regenerate in the greater part of the operative wound.

The areas of disintegrated bone or new growth tissue at the root ends of dead teeth can not be adequately treated through the root canals of the teeth. Hundreds of patients examined a few years after such treatment showed very clearly that the treatment was inadequate. Areas of disintegrated bone can not be drained and cured through the root canals of the dead teeth.

The old method of "pulling" or extracting dead teeth in which putrefactive changes have taken place, commonly, will not suffice to remove all of the diseased area. Blind curetting after extraction may be helpful or it may be harmful, depending on whether or not all septic granulations and disintegrated bone are removed. I have seen many serious results following incomplete removal of such structure. Blind curetting is at best uncertain, and the location of septic areas are often such that curetting can not reach them without opening of the parts to direct access and vision. The free sweep of the curette in the deeper pathological cavity is impossible in many cases because the upper part of the septum separating the roots is still intact.

When dead teeth are extracted septic tissue is often retained in the jaws. Broken down alveolar process and granulations, unseen by the dentist who "pulls" the tooth may cause systemic toxemias or bacteremias. In fact, the septic tissue retained after a single putrescent tooth is extracted may cause infective thrombosis and embolism following the opening of the facial veins and results in death.

Moreover, the dentist who extracts teeth will probably fail to discover such complications as antral perforations, necrotic antral walls, polypi, and pathological involvement of the canals which supply the nutrient arteries and nerves of the jaws.

Frequently after the extraction of a putrescent tooth the necrotic structure which is not removed causes a chronic low grade suppuration. This goes on perhaps for a number of years and causes extensive loss of alveolar process.

Before putrescent teeth are "pulled" diagnosis by means of roentgenograms is not difficult. After the "pulling," if septic tissue remains in the jaw, a low grade suppuration may go on for several years unknown to the patient. When finally a heart lesion, a rheumatic joint, or some other direct result of the suppuration becomes apparent, diagnosis is extremely difficult. A roentgenogram may not indicate clearly the condition in the jaws because more or less eburnation has taken place in the alveolar process surrounding the septic area. Diagnosis can be made only by exploratory operation. Frequently diagnosis is too late, because in case of heart lesion, for instance, while the cause of the trouble may be removed from the jaw, the damage to the heart itself can not be removed.

We have attempted to show why dead teeth in which putrefactive changes have taken place should be dissected out, not "pulled" or extracted.

It is natural now to ask whether it is possible to retain in the jaws dead teeth in which putrefactive changes will not take place. Various methods have been devised with the aim of sterilizing dead dentin so as to prevent putrefactive and suppurative changes from taking place in the tooth and the surrounding parts. The efforts of dentists today, in fact, seem to be directed toward the "treatment" of dead teeth more than toward any other problem.

When the vital pulp has been removed from a tooth its blood supply has been cut off; and the tooth dentin dies. The dead tooth dentin containing approximately twenty-eight per cent. of organic matter is subject to the laws of decomposition. Deleterious changes in this dead organic matter may be retarded. An area in the jaw filled with fibrous granulations and different strains of micrococci may be rendered passive so far as acute symptoms are concerned. Absorption may be temporarily arrested from such an area and putrefactive changes may be retarded in the tooth itself.

But a few years after a tooth has been devitalized it will show evidence of putrefactive changes. I have examined hundreds of dead teeth treated by all the known methods of sterilization and embalming; and I have failed to find a single one which did not show clear evidence of putrefactive changes some years after devitalization. The color and the odor of the opened tooth would be enough to convince any one of the failure of the "treatment" and "cure."

Therefore we may conclude that every devitalized tooth will in course of time become putrescent. And since this is true we must add that no tooth should be devitalized. The free devitalization of teeth is almost criminal.

When devitalization can not be prevented, putrefactive changes are bound to take place in the tooth itself and probably in the surrounding parts.

Immediately after devitalization only, may a dead tooth be extracted by the old method without danger to the patient. The dentist who finds it absolutely necessary to devitalize the tooth should extract the tooth immediately.

But if the devitalized tooth has remained in the jaw until putrefactive changes have taken place in it and probably in the surrounding parts, a roentgenogram may offer evidence of this. There may be drainage ways so minute that the roentgenogram will not disclose them. There may be perforations into neighboring cavities or canals which blind probing will not indicate. The cancellous bone may take up septic exudate.

Extraction in such cases will never offer a certain "cure." The cure demands the removal of dead teeth, necrosed bone, and pyogenic granulations thoroughly. The thorough removal is possible only by means of the surgical dissection outlined above.

